# Work environment factors and provider performance in health houses: a case study of a developing country

**DOI:** 10.1186/s13104-020-05346-1

**Published:** 2020-10-27

**Authors:** Hasan Yusefzadeh, Bahram Nabilou

**Affiliations:** 1 Department of Management and Health Economics, School of Public Health, Urmia University of Medical Sciences, Urmia, Iran; 2 Department of Management and Health Economics, School of Public Health, Urmia University of Medical Sciences, Nazloo Paradise, Sero Road, Urmia, West Azerbaijan 575611611 Iran

**Keywords:** Equipment layout, Performance, Community Health Worker, Urmia

## Abstract

**Objective:**

Primary Health Care has determined the path to the goal of "Health for All". Defining standards in health facilities play a crucial role in achieving acceptable performance by Community Health Workers. The study aimed to assess the relationship between physical Work environment factors and performance in primary healthcare facilities named health houses in Urmia district health network in North West of Iran. Thirty-five health houses were selected and studied with simple random sampling method. Data collection instrument were a standard checklist.

**Results:**

The results highlighted a statistically significant and positive correlation between technical equipment layout (P = 0.01, r = 0.641) with the performance of CHWs and the area of workplace (P = 0.05, r = 0.359) in health houses. Correlation between office equipment layout and performance was negative (P = 0.01, r = − 0.44). Multiple linear regression analysis showed that the performance level was influenced by the staff-mix of CHWs in health houses, layout of technical equipment and layout of office equipment**.**

## Introduction

Better health outcomes, improved access to health care, efficiency, and users’ satisfaction are the features of the health systems with a Primary Health Care (PHC) approach, which determined the path to the goal of “Health for All” [1]. Member states of the World Health Organization (WHO) agreed to PHC policy in 1978 and in the last decade have been invited by WHO to revive the PHC [2].

In this regard Iran has gained a position in providing PHC services through District Health Networks. These networks cover above 18,000 health houses and 6500 urban and rural health centers across the country [3, 4]. Health houses have been known responsible for improving health indicators in Iran [5, 6].

Community Health Workers (CHWs) called Behvarz, manage a health house as the most peripheral state facility in Iran [7, 8]. CHWs are in charge of promotional, preventive and curative tasks [8], including census, health education, maternal and child health, reproductive health, management of communicable and non-communicable diseases, immunization, and so on [9, 10].

Some studies have demonstrated the effectiveness of CHWs in delivering key health interventions [11]. Good outcomes for patients depend on the competent and sufficient number of healthcare workers with appropriate resources [12].

The "environment" has three broad areas: people, culture, and physical space [13]. Physical space is one of the main expenses in the organization [14] and there is a lot of research on the impact of physical work environment factors on positive organizational outcomes [15]. Several factors in the environment like workplace design directly or indirectly affect employee work performance and productivity [16].

There is an association between work environment factors (WEFs) and the performance of CHWs [17].

The work environment have an important role in the performance of a health worker [18]. Some studies reported the impact of physical environment on better coverage of health services [19] or effectiveness and efficiency of the health workers [20].

Because of its positive impact on more efficient performance, improvement in the layout of the workplace has been noticed in health care settings [21–23]. Availability of workspace and equipment has a significant effect on the performance of health workers as two elements of the working environment [24].

Studies suggest little research has been conducted on the health system factors [8, 25] affecting CHWs performance in developing countries, especially Iran [26]. In a previous study by the author, results suggested a positive correlation between the layout of technical equipment on performance in health houses [27].

Therefore, this study aimed to determine the correlation between the physical components of WEFs with the performance of health houses affiliated with the Urmia District Health Network (UDHN). Physical components included specifications of the area of health houses and the layout of equipment [24].

## Main text

### Study background

This study is a secondary data analysis of the cross-sectional observational study was conducted in health houses affiliated with UDHN, the capital city of West Azerbaijan Province, in the northwest of Iran, from January to April 2014.

The sample size was determined to be 35 health houses. The study samples were proportionally selected with stratified random sampling method from among 196 health houses regarding eight outgoing routes from the Urmia city.

### Data collection

Data collection was performed using a valid and reliable instrument including four subscales: (1) background and demographic information of health houses and health workers, (2) checklist of area workspace in health houses, (3) performance checklist and (4) checklist of equipment layout.

To assess the performance, checklists were selected regarding nine service delivery programs in health houses. Service delivery programs included the maternal health, reproductive health, management of communicable diseases, management of non-communicable diseases, vaccination, healthy child, integrated management of childhood illness, basic first aid and treatment of simple symptoms.

Trainers of Behvarz Training Centre (BTC) observed and completed performance checklists in all of the selected health houses [28].

A maximum score of 10 was determined both for layout of the technical equipment and office equipment for full compliance with the standards. Items of layout checklists were graded as yes = 1/no = 0 to assess the observance with standards.

In regard with the area of workspace a maximum score of 10 was determined for the full observance of the standards related to the number of the rooms and area including examination room, workroom and waiting room (2 + 2 + 2 scores) based on the presence or absence of the room (yes = 2 and no = 0). The size of the entire area of workspace got a maximum score of 4 in the health houses. Data about the entire area of the health houses was obtained from the documents available in Urmia District Health Center.

### Statistical analysis

SPSS software (version16) used for data analysis. The researchers applied the Shapiro–Wilk test to assess normality of dependent variable distribution. Considering normal distribution for performance (P = 0.207), parametric tests were used for data analysis. The data analyzed by means of descriptive statistics methods (e.g. frequencies, and percentages) and analytical methods (e.g. Pearson correlation coefficient test for determining correlation among independent variables and performance; independent T-Test for staff-mix in the health houses and numbers of CHWs in health houses; and multiple linear regression). Internal reliability of checklists evaluated with Crombach's alpha (α = 0.840). In all statistical analyses P < 0.05 was considered significant.

## Results

In this study 35 health houses were investigated. The staff of the health houses included 65 CHWs (Behvarz). Maximum and minimum population covered by a health house were 2963 and 487 persons, respectively. The age of the health houses buildings ranged from 2 to 41 years (Additional file 1: Table S1).

Scores of layout and workspace calculated in health houses. Mean scores of layout of technical equipment was higher than office equipment (Table 1).

**Table 1:** Mean, maximum, minimum and SD of scores of layout and workspace in studied health houses (N = 35)

NO	Variable	Minimum	Maximum	Mean	SD
1	Area of workspace	4.55	10.00	8.21	1.48
2	Layout of technical equipment	7.75	9.35	8.65	0.45
3	Layout of office equipment	6.00	7.75	6.90	0.43
4	Performance	7.17	9.35	8.64	0.50

The sex composition of CHWs in the health houses included two types: only female (n = 7) and both sexes together (n = 28). Independent T-Test showed no statistically significant difference between two type of sex composition, based on the performance scores (P = 0.954), and the layout scores (P = 0.305).

Association between the numbers of CHWs in health houses (one or two persons) and performance was also investigated. Independent T-Test results showed no statistically significant difference (P = 0.979).

A positive correlation was shown not only between layout of technical equipment and performance (P = 0.00, r = 0. 641) but also between the area of workspace and performance (P = 0.049, r = 0.366). Meanwhile, there was a negative correlation between the layout of office equipment and performance (P = 0.01, r = − 0.44) (Table 2).

**Table 2:** Correlation between dependent and independent variables

NO	Variables	1	2	3	4	5	6	7	8	9
1	Building lifetime	1								
2	Population	0.077	1							
3	Distance from City	− 0.359*	− 0.097	1						
4	Number of staff	0.298	0.415*	− 0.131	1					
5	Number of rooms	− 0.206	− 0.135	0.133	− 0.237	1				
6	Area of workspace	− 0.056	0.214	− 0.025	0.004	0.757**	1			
7	Technical layout	0.137	0.189	− 0.167	0.244	0.081	0.359*	1		
8	Office layout	− 0.434**	− 0.360*	0.363*	− 0.315	0.201	− 0.123	− 0.315	1	
9	Performance	0.181	0.185	− 0.294	− 0.018	0.010	0.336*	0.641**	− 0.440**	1

**Significant at 0.01, *Significant at 0.05

Backward method multiple linear regression was used to model the relationship between explanatory variables including age of the building, ownership, distance from the city, number of staff, number of rooms, the area of workspace and layout of equipment with performance as the response variable. Results showed that the performance was influenced by the staff-mix of CHWs in health houses, layout of technical equipment and layout of office equipment (Table 3). Therefore the final model was as follows:

$${\text{Y}} = {6}.00{5} - 0.{3}0{\text{9X}}_{{1}} + 0.{\text{646X}}_{{2}} - 0.{\text{417X}}_{{3}}$$

Y = Performance, X_1_ = Staff-mix of CHWs in health houses, X_2_ = Layout of technical equipment, X_3_ = Layout of office equipment.

**Table 3:** Multiple linear regression analysis on performance of health houses (n = 35)

NO	Independent variables	Standardized coefficients (Beta)	Unstandardized coefficients (CI 95%)	T value	P value
1	(Constant)	–	6.479	3.379	0.002
2	Sex composition HCWs	− 0.275	− 0.309 (− 0.614 to − 0.005)	− 2.072	0.047
3	Layout of technical equipment	0.580	0.646 (0.352–0.939)	4.488	0.000
4	Layout of office equipment	− 0.362	− 0.417 (− 0.739 to − 0.095)	− 2.639	0.013

## Discussion

Based on published materials about WEFs and the performance in health facilities, the current research was one of the few studies conducted on the performance of health houses in Iran [26, 27]. This study showed the effect of physical work environment on CHWs‟ performance in UHDN.

A good infrastructure is effective in improving employees’ performance [29–31]. In studies on the performance and productivity of organization including health systems, WEFs such as workspace and equipment have been highlighted [32–35]. Several studies have also examined the equipment layout [21–23].

The results of the study are consistent with those of Musembi [36] who reported the impact of work environment on the staff productivity [37].

Discomfort at the workplace cause health problems in employees, which lead to increased absenteeism and decreased productivity [38]. This confirms the results of [37] who reported that optimum position is achieved by physical environment.

Top has introduced WEFs among the most influential factors in the performance of nurses [34]. In this study, the positive correlation was observed between the layout of technical equipment and the performance.

The results of the present study are also in line with what Sehgal reported as designing a workplace is an inevitable factor in improving the performance of individuals, even emphasizing the specific role of every component in the performance [35]. Proper layout reduces extra movements to get the job done, increases the efficiency, and finally improves the performance [39].

Maji has reported an association between the size of workspace dedicated to the provision of services and more vaccination coverage which was not consistent with the results of the present study [19]. Fort, in his study in Armenia, found that workspace, equipment, and organization of work were important factors in the performance of staff and that the workspace was an important element from employees' perspectives [40]. The relevance and importance of workspace and layout of equipment were revealed in the present study, too. There was a positive correlation among performance, workspace and technical layout in this study.

The physical environment factors contribute reducing errors; increasing comfort; and enhancing control. Features like wayfinding is a practical action that improve the health worker conditions in healthcare facilities [41].

According to Oswald, such characteristics of work environment as workspace and equipment have a considerable effect on the performance of health workers in the reproductive and child health unit [24] and the study by Sadatsafavi showed the effect of physical work environment (including building layout, furniture, and finishing materials) and health human resource practices on each other and this confirms the results of the present study regarding layout [42].

Personal workspace was ranked as the most important feature of the physical environment that satisfies employees [43]. Findings of Kanamori indicated that improvement in service quality was the result of changes in the workplace including layout [23] and Young concluded that consideration of different aspects of layout resulted in efficient workplaces with enhanced safety and increased productivity [21]. The results of the present study are in line with the above-mentioned findings.

Asigele indicated that the working environment factors such as space and equipment have an effect on the performance of CHWs [24]. Service delivery in front line units like health houses required that CHWs must be equipped with technical skills and instruments to perform efficiently [32].

One study in Pakistan showed a positive relationship between the performance of employees [44]. The present study showed a positive correlation among the layout of technical equipment, workspace and performance and it was negative about office equipment.

All of the office equipment and technical equipment are arranged on the same desk in studied health houses. Office equipment can make it harder to access technical equipment and impede the provision of services. This issue could explain the negative correlation between the layout of these items and the performance.

In the health houses with more than one CHW, the male one is responsible for activities that are mostly done outside of health houses such as making follow-up visits and environmental health [9]. These activities were not included in this study. In other words, layout and workspace were studied in relation to indoors activities in health houses which was done by female CHW. In such a situation, attribution of the performance of the health house to two CHWs confuses the results.

## Conclusion

At the operational levels such as health houses, service provision is not possible without equipment that must have been arranged with a special order. Sufficient and standard workspace is a necessity for proper layout. Therefore, improvement in performance and productivity requires much attention to be paid to the WEFs. The release of standards of layout and workspace for health houses by national authorities also highlights the importance of this issue in Iran.

## Limitations

One of the limitations of this study was the lack of sufficient studies conducted on the effect of WEFs on the performance of employees in health facilities, and thus, cautious interpretation is advised. Equal weighting of programs in performance assessment and allocation of equal scores to different pieces of equipment may be considered as one of the limitations.

## Supplementary information

**Additional file 1: Table S1.** Mean, maximum, minimum and SD of scores of layout and workspace in studied health houses (N = 35).

## Data Availability

The datasets used and/or analysed during the current study are available from the corresponding author on reasonable request.

## Abbreviations

CHWs: Community Health Workers
WEFs: Work environment factors
PHC: Primary Health Care
WHO: World Health Organization
UDHN: Urmia District Health Network
BTC: Behvarz Training Centre
