# Supplementary material for: Work environment factors and provider performance in health houses: a case study of a developing country
Source: BMC Res Notes. 2020 Oct 27;13:498. doi: 10.1186/s13104-020-05346-1 (PMC7590718; doi:10.1186/s13104-020-05346-1)
Supplement: Supplementary file 1 — Additional file 1: Table S1. Mean, maximum, minimum and SD of scores of layout and workspace in studied health houses (N = 35). [file 13104_2020_5346_MOESM1_ESM.docx]

Table S1: Mean, maximum, minimum and SD of scores of layout and workspace in studied health houses (N = 35)

| NO | | Variable | Minimum | Maximum | Mean | SD |
| --- | --- | --- | --- | --- | --- | --- |
| 1 | Area of workspace | | 4.55 | 10.00 | 8.21 | 1.48 |
| 2 | Layout of technical equipment | | 7.75 | 9.35 | 8.65 | 0.45 |
| 3 | Layout of office equipment | | 6.00 | 7.75 | 6.90 | 0.43 |
| 4 | Performance | | 7.17 | 9.35 | 8.64 | 0.50 |
